# Recent trends in cardiovascular disease deaths: a state specific perspective

**DOI:** 10.1186/s12889-021-11072-5

**Published:** 2021-06-01

**Authors:** Sheila M. Manemann, Yariv Gerber, Suzette J. Bielinski, Alanna M. Chamberlain, Karen L. Margolis, Susan A. Weston, Jill M. Killian, Véronique L. Roger

**Affiliations:** 1 Department of Quantitative Health Sciences, Mayo Clinic, 200 First Street, SW, Rochester, MN 55905 USA; 2 Department of Epidemiology and Preventive Medicine, School of Public Health, Sackler Faculty of Medicine, Tel Aviv University, Tel Aviv, Israel; 3 HealthPartners Institute, Minneapolis, MN USA; 4 Department of Cardiovascular Diseases Medicine, Mayo Clinic, Rochester, MN USA; 5 Epidemiology and Community Health Branch, National Heart, Lung and Blood Institute, National Institutes of Health, Bethesda, MD USA

**Keywords:** Community surveillance, Cardiovascular disease, Epidemiology, Secular trend analysis

## Abstract

**Background:**

The rate of decline in cardiovascular disease (CVD) mortality has lessened nationally. How these findings apply to specific states or causes of CVD deaths is not known. Examining these trends at the state level is important to plan local interventions.

**Methods:**

We analyzed CVD mortality trends in Minnesota (MN) using the U.S. Centers for Disease Control and Prevention (CDC) Wide-ranging ONline Data for Epidemiologic Research (WONDER). Trends were analyzed by age, sex, type of CVD and location of death.

**Results:**

CVD mortality rates in MN declined in 2000–2009 and then leveled off in 2010–2018, paralleling national rates. Age- and sex-adjusted CVD mortality decreased by 3.7% per year in 2000–2009 (average annual percent changes [AAPC]: -3.7; 95% CI: − 4.8, − 2.6) with no change observed in 2010–2018. Those aged 65–84 years had the most rapid early decline in CVD mortality (AAPC: -5.9, 95% CI: − 6.2, − 5.7) and had less improvement in 2010–2018 (AAPC: -1.8, 95% CI: − 2.2, − 1.5), and the younger age group (25–64 years) now experiences the most adverse trends (AAPC: 1.2, 95% CI: 0.7–1.8). Coronary heart disease (CHD) and cerebrovascular disease had the largest relative decreases in mortality in 2000–2009 (CHD AAPC: -5.2; 95% CI: − 6.5,-3.9; cerebrovascular disease AAPC: -4.4, 95% CI: − 5.2, − 3.6) with no change 2010–2018. Heart failure (HF)/cardiomyopathy followed similar trends with a 2.5% decrease (AAPC 95% CI: − 3.5, − 1.5) per year in 2000–2009 and no change in 2010–2018. Deaths from other CVD also decreased in the early time period (AAPC: -1.6, 95% CI: − 2.7, − 0.5) but increased in 2010–2018 (AAPC: 1.9, 95% CI: 0.5, 3.3). In- and out-of-hospital death rates improved in 2000–2009 with a slowing in improvement for in-hospital death and no further improvement for out-of-hospital death in 2010–2018.

**Conclusion:**

Concerning CVD mortality trends occurred in MN. In the most recent decade (2010–2018) mortality from all CVD subtypes plateaued or even increased. CVD mortality among the younger age groups increased as well. These data are congruent with adverse national trends supporting their generalizability. These adverse trends underscore the urgent need for CVD prevention and treatment, as well as continued surveillance to assess progress at the state and national level.

## Introduction

In the United States (US), total cardiovascular disease (CVD) mortality declined steadily between the 1970s and 2016 [1, 2]. The rising prevalence of obesity and diabetes mellitus (type 1 and 2) over the same period suggested that if these risk factors had been controlled, gains would have been even greater. Since the turn of the century, the burden of obesity and diabetes mellitus escalated in the US [1, 3] and worldwide [4, 5], raising the concern that CVD mortality gains would eventually stop. Recent national data also suggested that blood pressure control steadily improved from 1988 to 2010, reached a plateau until 2016, and declined substantially in 2017–2018 [6]. Against the backdrop of these adverse risk factor trends, in 2011, the number of deaths from all CVD, heart disease, and stroke in the US began increasing, resulting in a slow down in the decline of corresponding mortality rates. The increase in absolute number and age-adjusted rates of deaths due to CVD is a significant event, which marks the time when we started losing ground in the fight against heart disease.

Partitioning of the causes of death revealed striking heterogeneity as the decline continued for ischemic heart disease, while age-adjusted rates of deaths from heart failure (HF) actually reversed from a downward to a rising trend [7]. These critically important reports call for further analyses to understand geographic variation, cause-specific deaths, location of death, and age-specific trends in CVD mortality [3, 7]. Indeed, whether national trends in CVD mortality extend to a state with historically favorable health trends like the state of Minnesota (MN) is yet to be determined [8]. This is particularly important to plan local interventions. Therefore, we undertook this study to examine trends in CVD death from 2000 to 2018, overall and by age, sex, type of CVD and location of death, in the state of MN.

## Methods

Cardiovascular mortality rates from 2000 to 2018 for the US and MN were obtained using the Centers for Disease Control and Prevention (CDC) Wide-Ranging ONline Data for Epidemiologic Research (WONDER) data set. This data source includes the assigned cause of death from all death certificates filed in the 50 states and the District of Columbia [9]. Underlying cause of death was categorized using ICD 10 codes for all CVD (I00-I99) deaths. Rates are available overall and by demographic subgroups (age and sex), CVD death subgroups (coronary heart disease (CHD), cerebrovascular disease, rhythm disorders, HF and cardiomyopathy, valvular disease, peripheral artery disease (PAD), venous thromboembolism (VTE), high blood pressure, and other), location (in-or out-of-hospital) and residence (urban vs. rural) at death.

Behavioral Risk Factor Surveillance System (BRFSS) data for MN was ascertained for self-reported obesity (body mass index, ≥30) among adults aged 18 and older [10]. The BRFSS is a state-based system of health surveys collecting information on health risk behaviors, preventive health practices, and health care access primarily related to chronic disease, and injury. The US Diabetes Surveillance System, an interactive web application that allows users to view diabetes surveillance data and trends at national, state, and county levels [11], was used to obtain self-reported diabetes mellitus (type 1 and 2) among adults aged 18 and older. This study was exempt from Institutional Review Board approval because all data were de-identified and publicly available.

### Statistical analyses

Yearly number of deaths and population counts, overall and by sex, age (3 categories: 25–64, 65–84, 85+), type of CVD death, location of death, and residence at death, were obtained among those aged 25 and older. Crude and age-adjusted (to the US 2010 population) yearly rates were also obtained except for rates by age groups where only crude rates were available. Overall and category-specific temporal trends in yearly death rates for the time periods 2000–2009 and 2010–2018, which correspond to time periods used in previous work [3], were explored using negative binomial regression, which enables estimates of sex- and age-adjusted (as appropriate) average annual percent changes (AAPCs). Differences in AAPCs between 2000 and 2009 and 2010–2018 were tested overall and across demographics, type of CVD death, and location of death categories. When examining trends by type of CVD death, due to the small number of deaths in certain groups, an ‘other CVD’ category was created that included, but was not limited to, rhythm disorders, valvular disease, PAD, VTE, high blood pressure. For all categories except age, age was adjusted for in the model as a categorical variable (25–64, 65–84, and ≥ 85). Analyses were performed using SAS statistical software, version 9.4 (SAS Institute Inc., Cary, NC).

## Results

**Fig. 1:**
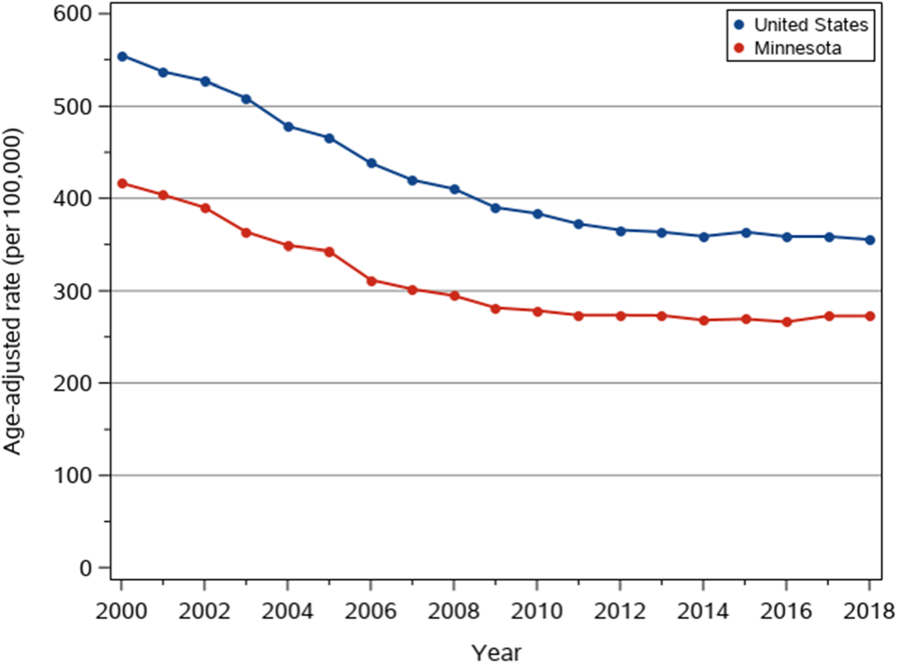
Trends in total cardiovascular mortality for United States and Minnesota, 2000–2018. Yearly rates per 100,000 persons have been standardized by the direct method to the age distribution of the U.S. 2010 total population

CVD mortality rates in MN declined in 2000–2009 and leveled off in 2010–2018, paralleling national rates (Fig. 1). The overall CVD mortality rate per 100,000 was 346 in 2000–2009 and it declined to 270 in 2010–2018 (Table 1). Age- and sex-adjusted CVD mortality decreased by 3.7% per year in 2000–2009 (AAPC: -3.7; 95% CI: − 4.8, − 2.6) with no change observed in 2010–2018 (AAPC: 0.0; 95% CI: − 1.5, 1.5; Table 1). Men had a higher rate of CVD mortality than women; however both groups had a similar decline in the rate of CVD mortality in 2000–2009 with no further improvement in 2010–2018 (Table 1). The rate of CVD mortality increased with age (Table 1), however those aged 65–84 had the most rapid decline in CVD mortality from 2000 to 2009 (AAPC: − 5.9, 95% CI: − 6.2, − 5.7), with a slowing of decline in 2010–2018 (AAPC: -1.8, 95% CI: − 2.2, − 1.5; Table 1). Those in the youngest (age 25–64) and oldest (age 85+) age groups also had a decrease in CVD mortality in 2000–2009 (Table 1). While those aged 85+ showed no change in CVD mortality in 2010–2018 (AAPC: 0.2, 95% CI: − 0.1, 0.5), those aged 25–64 experienced an increase (AAPC: 1.2, 95% CI: 0.7, 1.8).

**Table 1:** Cardiovascular disease mortality rates^a^ (95% CIs) and average annual percent change (AAPC)^b^ (95% CIs) in cardiovascular disease mortality rates among Minnesota residents from 2000 to 2018

Population	Time Period			
	2000–2009		2010–2018	
	Rate	AAPC	Rate	AAPC
Overall	346 (344–348)	-3.7 (− 4.8 to − 2.6)	270 (269–272)	0.0 (− 1.5 to + 1.5)
**Demographic Subgroups**				
Male	421 (418–425)	-3.7 (− 4.5 to − 2.9)	329 (326–332)	− 0.1 (− 1.2 to + 1.1)
Female	288 (286–291)	− 3.8 (− 4.8 to − 2.9)	221 (219–223)	+ 0.1 (− 1.2 to + 1.3)
Age 25–64	58 (57–59)	− 1.1 (− 1.5 to − 0.7)	57 (56–58)	+ 1.2 (+ 0.7 to + 1.8)
Age 65–84	894 (886–902)	− 5.9 (− 6.2 to − 5.7)	594 (588–600)	− 1.8 (− 2.2 to − 1.5)
Age 85+	5482 (5435–5529)	− 3.6 (− 3.8 to − 3.4)	4572 (4531–4613)	+ 0.2 (− 0.1 to + 0.5)
**CVD Categories**				
CHD	149 (147–150)	− 5.2 (− 6.5 to − 3.9)	103 (101–104)	− 1.3 (− 3.0 to + 0.5)
Cerebrovascular Disease	73 (72–74)	− 4.4 (− 5.2 to − 3.6)	54 (53–54)	−0.6 (− 1.7 to + 0.5)
Cardiomyopathy and Heart Failure	41 (41–42)	− 2.5 (− 3.5 to − 1.5)	34 (34–35)	− 0.5 (− 1.8 to + 0.8)
Other CVD^c^	83 (82–84)	− 1.6 (− 2.7 to − 0.5)	79 (79–81)	+ 1.9 (+ 0.5 to + 3.3)
**Location of Death**				
In-hospital	110 (109–111)	− 4.5 (−5.3 to − 3.8)	74 (73–75)	− 1.4 (− 2.5 to − 0.4)
Out-of-hospital	237 (235–238)	− 3.3 (− 4.7 to − 1.9)	196 (195–198)	+ 0.4 (− 1.5 to + 2.4)
**Residence at death**				
Rural	384 (381–388)	−3.3 (−4.5 to −2.1)	311 (307–314)	+ 0.3 (− 1.3 to + 1.9)
Urban	326 (323–328)	−3.8 (− 5.0 to − 2.7)	255 (253–257)	+ 0.1 (− 1.4 to + 1.7)

^a^ Yearly rates (95% CIs) per 100,000 persons have been standardized by the direct method to the age distribution of the U.S. 2010 total population

^b^ Adjusted for age, and sex. Models stratified by sex were adjusted for age; models stratified by age group were adjusted for sex

All *P* values ≤0.05 for difference in AAPC between 2000 and 2009 and 2010–2018

CHD coronary heart disease; CVD cardiovascular disease

^c^ Other CVD included, but was not limited to, rhythm disorders, valvular disease, PAD, VTE, and high blood pressure

**Fig. 2:**
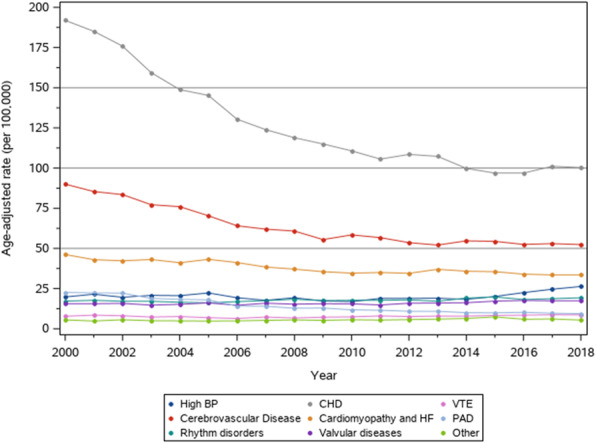
Trends in cardiovascular mortality subtypes for Minnesota, 2000–2018. Yearly rates per 100,000 persons have been standardized by the direct method to the age distribution of the U.S. 2010 total population BP blood pressure; CHD coronary heart disease; HF heart failure; PAD peripheral artery disease; VTE: venous thromboembolism

The largest proportion of CVD deaths was attributed to CHD (Fig. 2; 43% of CVD deaths in 2000–2009 and 37% in 2010–2018), followed by cerebrovascular disease (21 and 20% of CVD deaths, respectively) and HF/cardiomyopathy (12 and 13% of CVD deaths, respectively). CHD and cerebrovascular disease had the largest relative decreases in mortality in 2000–2009. Age- and sex-adjusted CHD mortality decreased by 5.2% per year in 2000–2009 (Table 1, AAPC: -5.2; 95% CI: − 6.5, − 3.9) with no change 2010–2018. Cerebrovascular disease decreased by 4.4% (AAPC 95% CI: − 5.2, − 3.6) per year in 2000–2009 and levelled off thereafter. HF/cardiomyopathy followed similar trends with a 2.5% (AAPC 95% CI: − 3.5, − 1.5) decrease per year in 2000–2009 and no change in 2010–2018. Deaths from other CVD also decreased in the early time period (AAPC: -1.6, 95% CI: − 2.7, − 0.5) but increased in 2010–2018 (AAPC: 1.9, 95% CI: 0.5, 3.3). In- and out-of-hospital CVD death rates improved in 2000–2009 with a slowing in improvement for in-hospital death and no further improvement for out-of-hospital death in 2010–2018 (Table 1). CVD death rates improved for both rural and urban residents from 2000 to 2009, however the rates leveled off in 2010–2018 (Table 1).

**Fig. 3:**
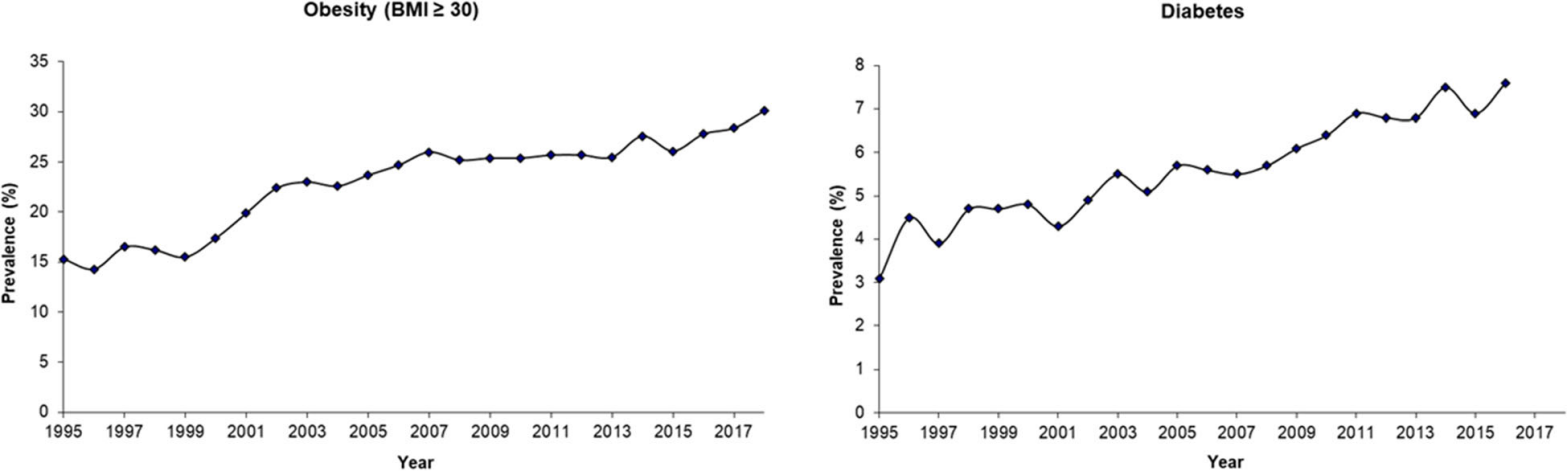
Prevalence of self-reported obesity and diabetes in Minnesota, 1995–2018. The obesity  data is from the Behavioral Risk Factor Surveillance System Survey and diabetes data from the US Diabetes Surveillance System

In MN between 1995 and 2018, the prevalence of diabetes mellitus and obesity increased among adults aged 18 and over (Fig. 3). The prevalence of obesity doubled over the study period, increasing from 15% in 1995 to 30% in 2018 and the prevalence of diabetes more than doubled, increasing from 3.1% in 1995 to 7.6% in 2016 (data not available after 2016).

## Discussion

In MN, CVD mortality trends have become quite unfavorable. In the earlier time period, CHD and cerebrovascular disease mortality rates had the largest decreases, but most recently mortality from all CVD subtypes plateaued or even increased. Mortality among the younger age groups increased as well. These data are congruent with recent national reports that CVD mortality in the US no longer follows the favorable decline observed since the mid-1960s [3, 7]. An analysis of deaths from ischemic heart disease, HF and other forms of heart disease uncovered that this national evolution was driven by increases in deaths related to HF and other forms of heart diseases [7]. Herein, we report that these unfavorable trends apply to MN, which had been historically ranked one of the top 10 healthiest states since 1990 according to America’s Health Rankings [8]. Although reports suggest that 30-day mortality rates after hospitalized MI have been declining [12, 13] and evidence-based treatment for MI has been increasing [14, 15], many CVD risk factors are also increasing. Obesity is prevalent [10] and diabetes mellitus rates are increasing, [11] and hypertension control is worsening [6], likely contributing to the slowing in decline of CVD mortality.

While the unfavorable CVD mortality trends are a concern in the US, in Europe recent mortality data do not support such a concern [16]. Available data from six European populations indicate that from 1985 to 2010 acute MI mortality decreased [17] and similarly, decreased from 2001 to 2014 in Norway [18], while in France CHD mortality decreased overall from 2006 to 2014 [19]. Adverse trends in risk factors, such as diabetes and obesity [4, 5], could however unfortunately lead to unfavorable CVD mortality trends as is currently observed in MN and the US.

While the trends in CVD mortality in MN did not differ by sex, they did so by age. Indeed, in recent years (2010–2018), CVD deaths increased among younger adults (age 25–64), a finding which is congruent with other reports [20–23]. Reasons for this unfavorable trend may include an increase in myocardial infarction incidence and in the prevalence of CVD risk factors among younger adults [21, 23, 24].

Furthermore, we found that while there was a decline in deaths in all of the CVD subtypes in the earlier time period, deaths from CHD, cerebrovascular disease, and cardiomyopathy/HF no longer declined in the most recent decade (2010–2018), possibly reflecting the increase in obesity and diabetes observed over the same period [10, 11]. Indeed these are all major risk factors for CHD, stroke, and HF [1]. It is notable that deaths in the other CVD category experienced an increase in recent years. However, because this group is heterogeneous it is difficult to draw conclusions, and this finding warrants further study. Taken collectively, these observations and the evidence that deaths from all CVD subtypes have either plateaued or increased, underscores the need for continued surveillance and emphasis on prevention and treatment of these conditions.

We also investigated location of death (in- vs. out-of-hospital) and residence at death (urban vs. rural). In- and out-of-hospital death rates improved in earlier years (2000–2009) with a slowing in improvement for in-hospital death and no further improvement for out-of-hospital death in recent years (2010–2018). Finally, CVD death rates initially improved for both urban and rural residents; however the rates leveled off in the recent time period.

We acknowledge that CDC WONDER data is code-based and subject to coding errors and although ICD-10 coding of the underlying cause of death was used the entire study period, we cannot rule out that changes in coding rules may have occurred. WONDER data also relies on underlying cause of death and we do not have information on other contributing factors of death. However, by using this national data source we can directly compare our results to the US overall and to other states. Our results emanate from the state of MN which has historically experienced favorable health rankings and is currently ranked the 7th healthiest state in the US [8]. Further, overall age- and sex-specific mortality trends are similar for MN and the entire US, and broad disease trends in MN are commensurate to national trends [25], supporting the broad relevance of our data.

We not only investigated overall trends in CVD mortality, but also investigated trends by age, sex, type of CVD, and location and residence at death. Furthermore, our report establishes CVD mortality trends prior to the coronavirus (COVID-19) pandemic. Recent reports in the US have indicated that hospitalizations for myocardial infarction as well as ST-segment elevation cardiac catheterizations have declined significantly since the COVID-19 pandemic began in early 2020 [26, 27]. In addition, the change in healthcare delivery due to COVID-19 may have impacted patients with HF [28, 29], and indeed a recent report from Denmark has indicated that the number of patients hospitalized with worsening HF or diagnosed with new-onset HF was reduced post COVID-19 [30]. The impact of COVID-19 on CVD mortality in the US will need to be studied, as one can hypothesize that it will have a substantial effect on trends. The present data serve to document the trends before the beginning of the pandemic.

## Conclusion

In MN, the CVD mortality rates are no longer declining, paralleling national trends. CHD and cerebrovascular disease mortality rates had the largest early (2000–2009) decreases. In the most recent decade (2010–2018) mortality from all CVD subtypes plateaued or even increased. Mortality among younger age groups increased as well. These findings call for renewed emphasis on CVD prevention and treatment, including local interventions. Continued surveillance will be critical to assess progress.

## Data Availability

The source of the mortality data for this study was the U.S. Centers for Disease Control and Prevention (CDC) Wide-ranging ONline Data for Epidemiologic Research (WONDER). https://wonder.cdc.gov/ The obesity prevalence data was obtained from the CDC Behavioral Risk Factor Surveillance System (BRFSS). https://www.cdc.gov/brfss/brfssprevalence/ The diabetes prevalence data was obtained from the CDC United States Diabetes Surveillance System. https://gis.cdc.gov/grasp/diabetes/DiabetesAtlas.html

## Abbreviations

AAPC: Average annual percent changes
BP: Blood pressure
BRFSS: Behavioral Risk Factor Surveillance System
CDC: U.S. Centers for Disease Control and Prevention
CHD: Coronary heart disease
CI: Confidence interval
CVD: Cardiovascular disease
HF: Heart failure
MI: Myocardial Infarction
MN: Minnesota
PAD: Peripheral artery disease
US: United States
VTE: Venous thromboembolism
WONDER: Wide-ranging ONline Data for Epidemiologic Research
